# ﻿Taxonomic reintroduction of *Taphrinaviridis* (Taphrinales, Ascomycota) associated with *Alnusalnobetula* as one of five well defined European species colonizing alders

**DOI:** 10.3897/mycokeys.108.127292

**Published:** 2024-09-10

**Authors:** Michaela Caboňová, Renáta Vadkertiová, Slavomír Adamčík, Kamila Bacigálová, Marek Slovák, Shanza Zaib, Miroslav Caboň

**Affiliations:** 1 Plant Science and Biodiversity Centre, Slovak Academy of Sciences, Dúbravská cesta 9, 845 23 Bratislava, Slovakia Plant Science and Biodiversity Centre, Slovak Academy of Sciences Bratislava Slovakia; 2 Culture Collection of Yeasts, Institute of Chemistry, Slovak Academy of Sciences, Dúbravská cesta 9, 845 38 Bratislava, Slovakia Institute of Chemistry, Slovak Academy of Sciences Bratislava Slovakia; 3 Department of Botany, Faculty of Natural Sciences, Comenius University in Bratislava, Révová 39, 811 02 Bratislava, Slovakia Comenius University in Bratislava Bratislava Slovakia; 4 Department of Botany, Charles University, Benátská 2, 128 01 Prague, Czech Republic Charles University Prague Czech Republic; 5 Department of Plant Pathology, University of Florida, 2527 Fifield Hall, 32611-0680 Gainesville, Florida, USA University of Florida Gainesville United States of America

**Keywords:** Alder, culture characterisation, *Exoascus*, identification key, morphology, taxonomy, witches’-brooms

## Abstract

Phylogenetic analysis of four DNA regions (ITS, LSU, mtSSU and *tef1α*) supported the existence of five European *Taphrina* species which colonise *Alnus* in Europe. In addition to previously well-defined species, *T.viridis* is, for the first time recognised, by molecular study as a species related to *T.sadebeckii*. Analysis of publicly available sequences of barcoding regions suggested that *T.viridis* is only associated with *A.alnobetula* and no other *Taphrina* species colonize this host tree. Symptomatic, morphological, and physiological characterisation of *T.viridis* are provided together with the key for identification of *Alnus* associated *Taphrina* species in Europe and North America.

## ﻿Introduction

*Taphrina* Fr. is a dimorphic fungal genus of Ascomycetes (Taphrinomycetes, Taphrinales), with a saprophytic yeast phase, and a parasitic mycelial phase that typically causes foliar lesions and deformities, inflorescence, and branch lesions (so-called witches’ brooms) on host plants (e.g. Kramer 1987; Inácio et al. 2004). More than a hundred currently accepted species on various host plants have been described worldwide (www.speciesfungorum.org accessed 28.3.2024). *Taphrina* species represent an intriguing subject for evolutionary and phylogenetic studies, due to their unique genomic characteristics, especially their gene contents which enable them to be both plant pathogens and saprophytes, but also relatively small genomes of 13 MB which contain low numbers of repeated elements and single copies of the rDNA (Cissé et al. 2013; Wang et al. 2020). Members of this genus are well-known for their narrow host specificity, growing mainly on the plant genera of the Betulaceae, Rosaceae and Salicaceae families (cf. Mix 1949; Bacigálová et al. 2003; Bacigálová 2010; Fonseca and Rodrigues 2011; Christita et al. 2022). Previous molecular phylogenetic analyses based on the ITS-LSU regions provided the first insights into the evolutionary history of *Taphrina*, for most morphologically defined taxa, confirming their host preference patterns (Rodrigues and Fonseca 2003; Petrýdesová et al. 2013, 2016; Selbmann et al. 2014).

This study focuses on selected *Taphrina* members parasitizing on alders (*Alnus*, Betulaceae). The tree genus *Alnus* is one of the main components of several riparian ecosystems in the temperate zone of the Northern Hemisphere, but also extends to Southern America (https://powo.science.kew.org/) and provides important ecosystem services (Douda et al. 2016; Alonso et al. 2021). *Taphrina* species are among the most important fungal infection agents in alders (Rohrs-Richey et al. 2011; Arhipova et al. 2011). Understanding the diversity and evolutionary relationships of individual *Taphrina* species that colonise *Alnus* is essential in order to elucidate those factors that determine their host specificity, geographic distribution, and pathogenicity. The five *Taphrina* species which parasitize the genus *Alnus* have been accepted in the current literature: *T.alni* (Berk and Broome) Gjaerum, *T.epiphylla* (Sadeb.) Sacc., *T.robinsoniana* Giesenh., *T.sadebeckii* Johanson and *T.tosquinetii* (Westend.) Tul., although more species have been recognised in the past (Mix 1949; Rodrigues and Fonseca 2003; Fonseca and Rodrigues 2011). The existence of these species was supported by previous phylogenetic studies which predominantly used sequences of ribosomal DNA; however, the sequences formed independent lineages, which indicates their polyphyletic nature (cf. Rodrigues and Fonseca 2003; Inácio et al. 2004; Selbmann et al. 2014; Petrýdesová et al. 2013, 2016). In addition to the above-mentioned species, several authors (Mix 1949; Bacigálová 1994, 2010) have recognised another species, *T.viridis* (Sadeb.) Maire which colonises *A.alnobetula* (= *A.viridis*) with a high mountain distribution. However, this *Taphrina* species has been defined so far only on the basis of its morphology and has not been confirmed genetically. *Taphrinaviridis* causes grey-yellow spots on alder leaves similar to those of *T.sadebeckii*, but can be morphologically and ecologically distinguished, as has been described in previous studies. *Taphrinaviridis* is restricted to *A.alnobetula*, while *T.sadebeckii* grows only on *A.glutinosa* and related alder species (Mix 1949; Bacigálová et al. 2003; Bacigálová 2010). In this study, we test the taxonomic status of *Taphrina* strains which colonises *A.alnobetula* to discern whether it represents a distinct species corresponding to the original definition of *T.viridis*. We aim to reconstruct the phylogenetic placement of the species and elucidate its relationships with other recognized alder-colonising *Taphrina* species by employing an expanded set of genetic markers comprising four distinct DNA loci, including low copy genes. In order to enhance our inquiry into the evolutionary dynamics, biodiversity and distribution of *Taphrina* species thriving on alder hosts, we have supplemented our investigation by incorporating environmental DNA-derived sequences encompassing the entire spectrum of *Taphrina* taxa associated with alders.

## ﻿Material and methods

### ﻿Sampling and strain isolation

We analysed five strains of *Taphrina* isolated from the infected leaves of *Alnusalnobetula* collected in the mountain forest zone of the Western Carpathians in Slovakia between 2013–2023. All the new strains were isolated from infected host plant tissues using the spore-fall method. The detailed isolation procedures, cultivation, storage and anatomical–morphological characterization followed Bacigálová et al. (2003). The isolates were deposited either in the Culture Collection at the Institute of Botany, Plant Science and Biodiversity Center, Slovak Academy of Sciences (BU) or in the Culture Collection of Yeasts in the Institute of Chemistry, Slovak Academy of Sciences (CCY). All of the yeast strains were stored at -70 °C in a liquid medium with 25% (v/v) glycerol. For the phylogenetic study, we used strains analysed by Fonseca and Rodrigues (2011), which also includes ex-type strains of *T.alni*, *T.epiphylla*, *T.robinsoniana*, *T.sadebeckii* and *T.tosquinetii*. Three additional samples of *T.sadebeckii* isolated from *A.glutinosa* were also included in the dataset. Following the results of previous studies (Rodrigues and Fonseca 2003), *T.populina* and *T.populi-salicis* were selected as an outgroup for multilocus analyses. For sampling details see Table 1.

**Table 1.** List of strains used for multilocus analysis with collection details and GenBank numbers of corresponding DNA sequences. Strains highlighted in bold represent ex-type collections. Accession numbers of newly generated sequences start with letters PP- or PQ-.

Species	Strain	Country	Host	ITS	LSU	*mtSSU*	*Tef1a*
*T.tosquinetii*	HA 1335	Slovakia: Kokava nad Rimavicou	*A.glutinosa*	PQ013119	AF492067	–	PP997880
*T.tosquinetii*	**CBS 276.28 (=HA 1314)**	Austria	*A.glutinosa*	PQ013120	PQ013136	KU134826	PP997881
*T.alni*	HA 1364	Slovakia: Biele Vody Valley	*A.incana*	PQ013121	AF492024	PQ013150	PP997882
*T.alni*	**CBS 683.93 (=HA 872)**	Austria: Falbeson	*A.incana*	PQ013122	PQ013137	KU134812	PP997883
*T.robinsoniana*	**CBS 382.39 (=HA 850)**	unknown	*A.incana*	PQ013123	PQ013138	KU134824	PP997884
*T.viridis*	SAV (BU TA4)	Slovakia: Žiarska dolina valley	*A.alnobetula*	PQ013124	PQ013139	PQ013151	PP997885
*T.viridis*	SAV (BU 095) (= CCY 58-9-2)	Slovakia: Žiarska dolina valley	*A.alnobetula*	PQ013125	PQ013140	PQ013152	PP997886
*T.viridis*	SAV (BU TA8)	Slovakia: Žiarska dolina valley	*A.alnobetula*	PQ013126	PQ013141	PQ013153	PP997887
*T.viridis*	SAV (BU TA9)	Slovakia: Žiarska dolina valley	*A.alnobetula*	PQ013127	PQ013142	PQ013154	PP997888
*T.viridis*	**SAV (BU 094) (=CCY 58-9-1)**	Slovakia: Žiarska dolina valley	*A.alnobetula*	PQ013128	PQ013143	PQ013155	PP997889
*T.sadebeckii*	SAV (BU R001) (=CCY 58-9-3)	Romania: Gilau	*A.glutinosa*	PQ013129	PQ013144	PQ013156	PP997890
*T.sadebeckii*	SAV (BU R002) (=CCY 58-9-4)	Romania: Gilau	*A.glutinosa*	PQ013130	PQ013145	PQ013157	PP997891
*T.sadebeckii*	SAV (BU R003) (=CCY 58-9-5)	Romania: Gilau	*A.glutinosa*	PQ013131	PQ013146	PQ013158	PP997892
*T.sadebeckii*	**CBS 102170 (=HA 1308)**	Germany: Oberpfalz	*A.glutinosa*	PQ013132	PQ013147	KU134825	PP997893
*T.epiphylla*	**CBS 111109 (=HA 1439)**	Slovakia: Belianske Tatry Mts.	*A.incana*	PQ013133	AF492039	KU134818	PP997894
*T.populi-salicis*	**CBS 419.54**	USA: California, Palo Alto	*Populustrichocarpa*	PQ013135	PQ013148	KU134822	PP997895
*T.populina*	**CBS 337.55**	Unknown	*Populusnigra*	PQ013134	PQ013149	KU134821	PP997896

### ﻿DNA isolation and sequencing

Genomic DNA was isolated from the cultures grown on yeast-peptone-dextrose (YPD) agar plates using an E.Z.N.A. Fungal DNA Mini Kit (Omega), following the manufacturer´s recommendations, with a prolonged incubation time, as described in Caboň et al. (2019). The amplification conditions followed the protocols published by Petrýdesová et al. (2013) and Kiran et al. (2021) and targeted four regions: (I) the internal transcribed spacer regions of nuclear ribosomal DNA (ITS) using the primers ITS5, ITS4 (White et al. 1990); (II) the partial large subunit of nuclear ribosomal DNA (LSU) with the primers LR5, LR0R (Vilgalys and Hester 1990); (III) the partial mitochondrial small rRNA subunit (mtSSU) with the primers SSU1, SSU2 (Sulo et al. 2009); (IV) part of the translation elongation factor 1-alpha (tef1α) with primers 983F, 1953R (Rehner and Buckley 2005). The PCR products were purified using Exo-Sap enzymes (Thermo FisherScientific, Wilmington, Germany) and sequenced at the SeqMe sequencing company (Dobříš, Czech Republic). All newly generated sequences were deposited in GenBank and their accession numbers are listed in Table 1.

### ﻿Phylogenetic analyses

Raw sequences were edited with Geneious version R10 (Kearse et al. 2012). Intra-individual polymorphic sites with more than one signal were marked with IUPAC ambiguity codes. All four single-locus datasets were aligned using the MAFFT on-line service (Katoh et al. 2019) using the version MAFFT 7 with the E-INS-I strategy (Katoh and Standley 2013), manually improved in Geneious version R10 (Kearse et al. 2012), and concatenated into one multilocus dataset using SeaView version 4.5.1 (Gouy et al. 2010). The resulting alignment was further analysed using the CIPRES Science Gateway (Miller et al. 2010) with two different methods: Bayesian inference (BI) and Maximum Likelihood (ML). For the ML analyses, the concatenated alignments were uploaded as FASTA files and analysed using RAXMLRAxML-HPC2 on XSEDE (8.2.12) (Stamatakis 2014) as a partitioned dataset under the GTR + GAMMA model with 1000 bootstrap iterations. For the BI analysis, the dataset was divided into four partitions: ITS, LSU, mtSSU and *tef1a*. The best substitution model for each partition was computed jointly in PartitionFinder 1.1.1 (Lanfear et al. 2012). The aligned FASTA datasets were converted to the Nexus format using Mesquite 3.61 (Maddison and Maddison 2019) and further analysed using MrBayes 3.2.6 7a (Ronquist et al. 2012) on XSEDE under following substitution models: GTR+I+G for ITS, HKY+I for LSU and mtSSU and SYM+I+G for *tef1a*. Bayesian runs (BI) were computed independently, twice, with four MCMC chains for 10 million generations until the standard deviation of split frequencies fell below the 0.01 threshold. The convergence of runs was visually assessed using the trace function in Tracer 1.6 (Rambaut et al. 2014).

### ﻿Analyses of species diversity and distribution using public databases and environmental DNA

To obtain a more comprehensive insight into the diversity and geographic distribution of the *Taphrina* species on alders, we searched both global databases, UNITE and Genbank, for ITS sequences with a 97% threshold similarity for each alder-colonising species. Moreover, we searched for additional information on the distribution of *T.viridis* in the database GlobalFungi, which incorporates short ITS reads from metabarcoding datasets by querying sequences with 100% similarity to the sequence of *T.viridis* (BU 094). All of the sequences retrieved from the BLAST-search were downloaded and supplemented with our sequences (Table 1), as well as representative sequences of other *Taphrina* species that colonises various host plants. The final ITS dataset was aligned through the MAFFT on-line service (Katoh et al. 2019) using MAFFT 7 with E-INS-I strategy (Katoh and Standley 2013), and manually improved in Geneious version R10 (Kearse et al. 2012). An unrooted Maximum Likelihood phylogenetic tree was calculated using RAXMLRAxML-HPC2 on XSEDE (8.2.12) (Stamatakis 2014) under the GTR + GAMMA model with 1000 bootstrap iterations.

The resulting trees for both datasets were visualised and annotated with TreeGraph 2 (Stöver and Müller 2010) and graphically improved in CorelDRAW X5 (Ottawa, Canada).

### ﻿Morphological and biochemical characterisation of the strains

The morphological characteristics of the *T.viridis* strains analysed were determined using methods described by Kurtzman et al. (2011). The micromorphological characters were observed in dried material using a ZEISS AxioScope A1 with an attached AxioCam camera (both Carl Zeiss, Jena). All characters were observed and measured at 600× magnification after short staining with CottonBlue, with the exception of the spores which were observed and measured with an oil-immersion lens at a magnification of 1000× after the same staining. Statistics for the measurements of microscopic characteristics were based on an analysis of all the material available with a minimum of 30 measurements per specimen and per microscopic character. The range of measured values is expressed as the mean ± standard deviation.

The physiological and biochemical characteristics of the yeast cultures were examined using the methods described by Kurtzman et al. (2011). Assimilation tests were performed using liquid and solid yeast-carbon and yeast-nitrogen base media (Biolife, Milano, Italy). Assimilation on the solid media was performed using 24-well plates: the respective medium containing a carbon or nitrogen compound was inoculated with 5 μl of the yeast suspension (10^8^ cells ml^-1^). The yeasts were grown at their optimal temperature (20 °C) for 21 days. The carbon and nitrogen compounds (Merck, Germany) were tested in concentrations of 1%. The assimilation of nitrite was tested in a concentration of 0.25% KNO_2_. The yeasts were also inoculated on those media without carbon and nitrogen compounds (control).

In the liquid media, strains were cultivated in L-shaped tubes, with an initial concentration of 10^8^ cells ml^-1^. The cell biomass was measured by its absorbance (660 nm) at regular intervals for a period of 21 days. The absorbance of strains grown in the presence of carbon and nitrogen compounds was compared to that of strains grown in a solution without these substances (control). The carbon and nitrogen compounds were tested in concentrations of 1%. When a yeast culture exhibited weak growth, the carbon and nitrogen compounds were tested in a concentration of 0.5%. The assimilation of nitrite was tested only in a concentration of 0.25% KNO_2_.

## ﻿Results

### ﻿Phylogenetic analysis

**Figure 1.**
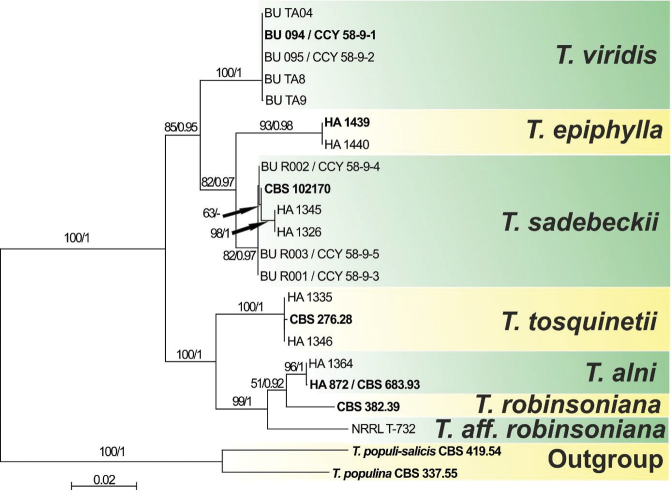
Phylogram generated by Maximum Likelihood (RAxML) analysis based on concatenated sequences of ITS, LSU, mtSSU and *tef1a* for the *Alnus*-colonising *Taphrina* species. Maximum likelihood bootstrap support values greater than 50% and Bayesian posterior probabilities greater or equal to 0.90 are indicated above or below the nodes. Sequences of type strains are highlighted in bold.

The final multilocus alignment consisted of 3485 positions, of which 736 positions, including gaps, belong to ITS, 851 positions to LSU, 957 to mtSSU and 941 to *tef1a*. Overall tree topologies of the ML and BI analysis were congruent (Fig. 1). The analyses revealed the presence of two strongly supported clades with two and three species-specific subclades, respectively. Sequences of the *Taphrinaviridis* strains isolated from *A.alnobetula* formed strongly supported subclade (ML=100, BI=1), placed as a sister group (ML=85, BI=0.95) to the subclade of *T.sadebeckii* and *T.epiphylla* (82/0.97). There is no support for any grouping of North American and European strains into a species rank lineage, but American strains labelled as *T.robinsoniana* and *T.aff.robinsoniana* are placed in the subclade together with European *T.alni*.

**Figure 2.**
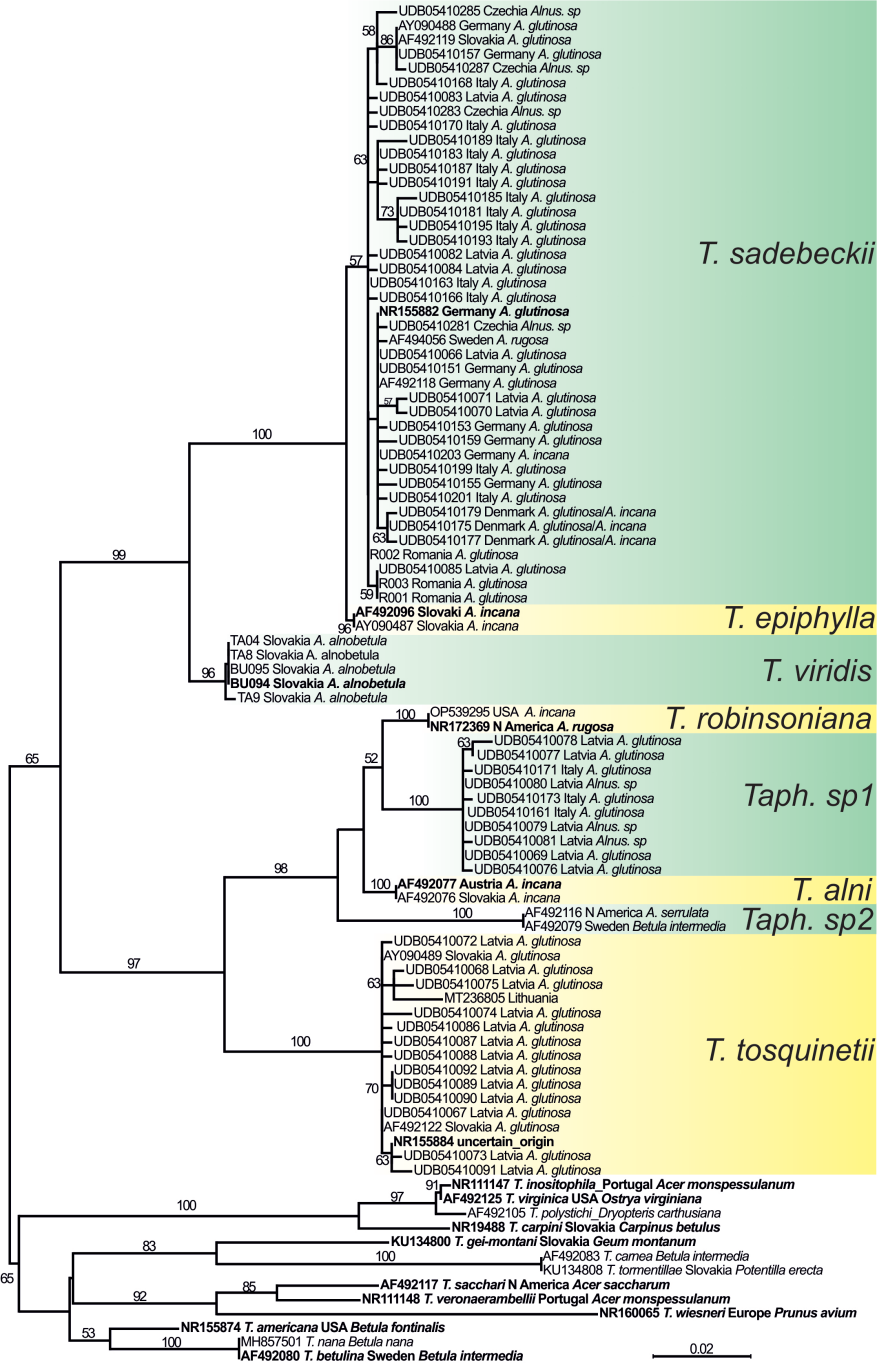
Unrooted phylogram generated by Maximum Likelihood (RAxML) analysis of ITS region combining the sequences originated from studied material, supplemented with additional sequences retrieved from BLAST Search. Bootstrap support values greater than 50% are indicated above branches. Sequences of type specimens are highlighted in bold.

For the ITS analysis of all the available sequences, the UNITE search retrieved an additional 62 ITS sequences which exhibited a high similarity of 97% to alder-colonising species. They were analysed together with 20 ITS sequences of strains used for multilocus analysis and 13 ITS sequences of other representatives of *Taphrina* species (Fig. 2).

Of the ITS sequences with high similarity analysed, almost all originated from *Alnus* samples, one ITS sequence came from the *Betula* sample. There is no additional data available for the type of *T.tosquinetii*. The highest number of sequences of alder-colonising species was retrieved for *T.sadebeckii* (36), followed by *T.tosquinetii* (14). No accessions identifiable with *T.viridis* or associated with *A.alnobetula* were recovered from the databases. All of the sequences originating from alder colonising species were clustered in a moderately supported monophyletic clade (BS=65). Likewise, in a multilocus analysis, there were two strongly supported clades of alder associated species with the same species clustering. Interestingly, the analysis revealed two strongly supported subclades (bootstrap support = 100 and 98) within the clade containing *T.alni* and *T.robinsoniana*, which included recently undiscovered *Taphrina* taxa. The first subclade consisted of ten samples from Latvia and Italy, isolated from *A.glutinosa*. They appeared in a sister position with *T.robinsoniana*, although this relationship demonstrated only weak statistical support. The second subclade was represented by two accessions, one isolated from *A.serrulata* in Sweden and the other parasitizing *Betulaintermedia* in North America.

### ﻿Taxonomy

#### ﻿ Taphrina viridis

Taxon classificationFungiTaphrinalesBetulaceae

(Sadeb.) Maire, Bull. Soc. Bot. Fr. 57: CLXVII (1912) [1910]

9D2AF5ED-0D15-5F7E-9006-8397DC89610C

537442

Exoascus viridis Sadeb. in Jaap, Deutsch. Bot. Monatsschr. 19: 76 (1901). Basionym.
= Taphrinaalnastri Lagerheim in Vestergren, Micr. Rar. Sel.: No. 720 (1903).

##### Neotype

**(here designated**; **MycoBank: MBT#10018607)**: • Slovakia; Vysoké Tatry Mts., Žiarska dolina valley; 1050 m a.s.l., on leaves of *Alnusalnobetula*; 31.7.2015; leg. K. Bacigálová; SAV (BU 094), deposited in herbarium at the Institute of Botany, Plant Science and Biodiversity Centre, Slovak Academy of Sciences (SAV).

##### Ex-type culture.

CCY58-9-1 (=SAV BU 094), deposited in Culture Collection of Yeasts, Institute of Chemistry, Slovak Academy of Sciences (CCY). Sequence accession numbers: ITSPQ013128, LSUPQ013143, mtSSUPQ013155, *tef1a*PP997889.

##### Symptoms in vivo.

**Figure 3.**
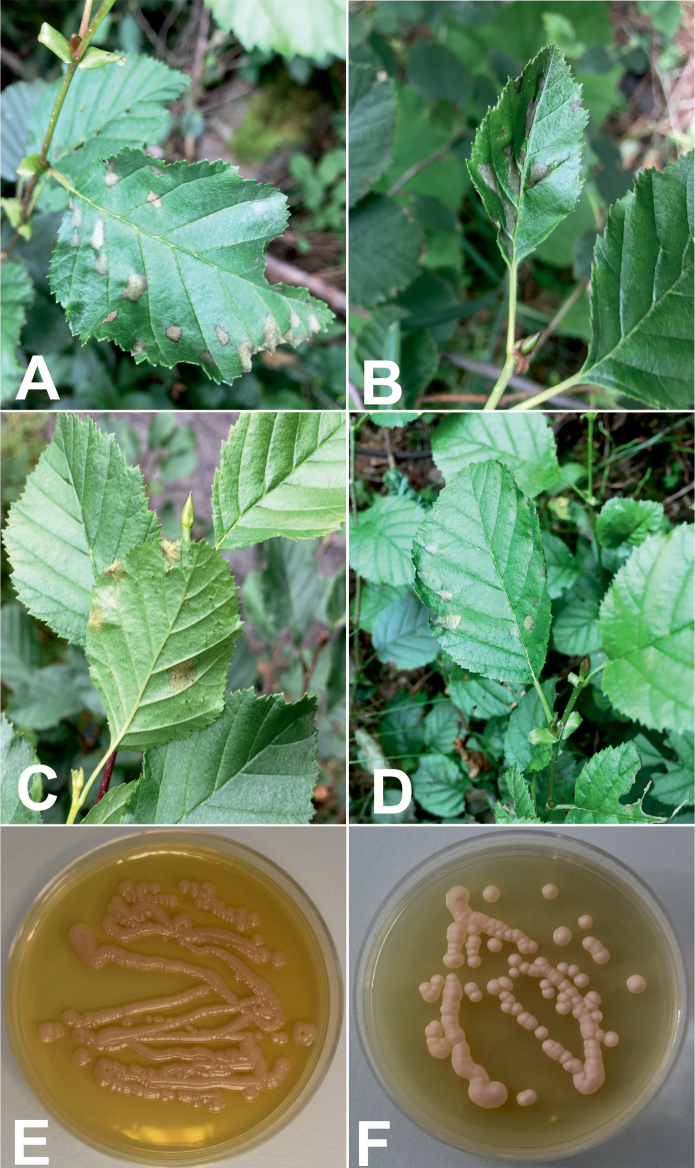
Macromorphological aspects of *Taphrinaviridis***A–D** field appearance and symptomatic **E, F** yeast cultures on yeast-peptone-dextrose agar (YPD).

*Taphrinaviridis* induces the development of small, rounded or irregularly shaped, pale green to yellow-green 5–10 mm large lesions on the upper or lower leaf surfaces of *Alnusalnobetula*, which in stage of mature asci become coated with a grey-white layer (Fig. 3A–D).

##### Description in vivo.

**Figure 4.**
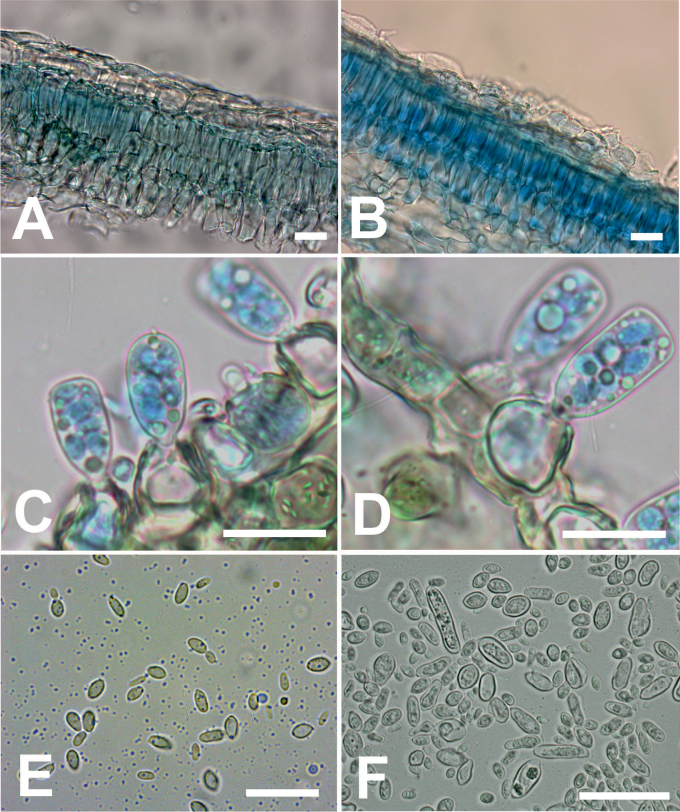
Micromorphological aspects of *Taphrinaviridis***A, B** subcuticullar vegetative mycelium **C, D** asci with ascospores and stalk cells **E** ascospores with post-release budding **F** yeast cells grown on yeast morphology medium at 20 °C for 6 days. Scale bars: 20 µm.

Vegetative mycelium grows subcuticulary in intercellular spaces of leaf tissues (Fig. 4A, B) and is composed of narrow, thick-walled cells, variable in length and shape, divided by layered septa, later mature into globose ascogenous cells. In the late stages of infection, ascogenous cells broaden and form asci, penetrating the epidermal layer of the leaf tissue. The asci are ovoid to ellipsoid, 21.9–24.5–27.1 × 11–11.8–12.8 μm (Fig. 4C, D). The apical parts of asci are mainly rounded or truncated with pale, translucent oil drops, the asci contain 8 spores. The ascospores are globose to ellipsoid, 5.3–6.5–7.7 × 3.9–4.4–4.9 μm (Fig. 4E); the ascospores budding post-release from asci, forms blastospores. Stalk cells are present, attached to asci, variable in shape and size, 12.4–14.8–17.3 × 11.1–14–16.9 μm.

##### Culture characteristics and physiological properties.

Colonies on the yeast morphology (YM) agar, after 21 days at 20 °C, are butyrous, smooth, slightly raised, with an even margin, creamy pink to pale pink colour (Fig. 3E, F); in a liquid yeast morphology medium after 6 days at 20 °C, the cells are ovoid to ellipsoidal, 2.5 – 9.9 × 5 – 9.9 μm, sometimes with buds; sometimes large single cells up to 16 μm (Fig. 4F); after 14 days at 20 °C, they form a sediment. Fermentation is absent. The assimilation of carbon compounds: d-glucose, sucrose, melezitose, d-xylose, cellobiose, glycerol, soluble starch, d-mannitol, and d-glucitol is positive. The assimilation of salicin and succinic acid assimilation is weak. Other carbon sources: maltose, lactose, raffinose, l-arabinose, inulin, trehalose, *myo*-inositol, melibiose, erythritol, rhamnose, galactose, ethanol, methanol, sorbose, d-arabinose, ribitol, lactate, d-ribose, xylitol, d-glukosamine, n-acetyl-d-glucosamine and citrate are not assimilated. The assimilation of nitrate, nitrite and l-lysine is positive; assimilation of ethylamine, creatinine and n-acetyl-d-glucosamine is negative. The urease reaction is positive; the diazonium blue B reaction is negative; the production of starch-like polysaccharide is positive. Growth on a vitamin-free medium is weak. Growth at 25 °C on yeast-peptone-dextrose agar is positive; at 28 °C negative.

##### Additional specimens examined.

• Slovakia; the Žiarska valley of the river Smrečianka; 1050 m a.s.l.; on the leaves of the host shrubs *Alnusalnobetula* along a touristic trail; 31.7.2015; leg. K. Bacigálová; BU 095 (SAV) / (CCY 58-9-2) • ibid.; 26.7.2013; leg. K. Bacigálová, J. Petrýdesová; BU TA4 (SAV) • ibid, 16.8.2022; leg. K. Bacigálová, G. Kozárová; BU TA8 (SAV) • ibid.; 17.8.2023; leg. K. Bacigálová, G. Kozárová; BU TA9 (SAV).

##### Note.

According to our phylogenetic analysis, *Taphrinaviridis* is related to *T.sadebeckii* and *T.epiphylla*. The symptoms of disease induced by these three species are similar: they cause yellow or grey lesions on alder leaves, which, in the case of *T.epiphylla* infections, may lead to the formation of witches’ brooms branch deformations (Table 2). In the field, *T.viridis* can be recognised by the more greenish and less greyish tinge of the lesions. Our data suggest that this species grows strictly on *Alnusalnobetula*, and no other *Taphrina* species has been recorded on this alder host tree. Under a microscope, *T.viridis* is characterised by a unique combination of long-narrow stalk cells (on avg. longer than 10 μm and narrower than 16 μm) and large ascospores (on avg. 6.5 × 4.4 μm), with post-release budding. The other *Alnus*-colonizing species, which has been reported with post-release budding, is *T.tosquinetii*; however it has distinctly smaller ascospores (Suppl. material 1). The physiological profile of *Taphrinaviridis* is very similar to that of the closely related *T.sadebeckii*. Both species utilise a broad range of carbon and nitrogen sources (see the species profiles), but *T.viridis* differs from *T.sadebeckii* in its ability to assimilate nitrite, its weak ability to assimilate salicin and succinic acid, and its inability to utilise inulin, xylitol and citrate.

**Table 2.** Review of host preferences and symptomatics of all detected *Alnus*-colonizing *Taphrina* species. Information retrieved from sequences originated from public databases are labelled as GenBank or UNITE, respectively.

Organism	Host preferences (*Alnus*)											Symptoms of disease	Reference
	*A.incana*	*A.glutinosa*	*A.×pubescens*	*A.hirsuta*	*A.hybrida*	*A.tinctoria*	*A.rubra*	*A.alnobetula*	*A.rugosa*	*A.serrulata*	*A.crispav.mollis*		
*T.alni*	+	+	+	+	+	+	+					Female catkins deformations	Mix 1949; Salata 1974; Bacigálová 2010
*T.epiphylla*	+											Witch broom, leaf curl, yellow or whitish-grey lesions	Mix 1949; Gjaerum 1964; Bacigálová 2010
*T.robinsoniana*	+								+	+		Female catkins deformations	Mix 1949; Mix 1954; GenBank
*T.sadebeckii*	+	+	+	+					+			Rounded, regular yellow lesions	Mix 1949; Salata 1974; Bacigálová 2010; UNITE; This study
*T.tosquinetii*		+	+		+						+	Leaf curl	Mix 1949; Gjaerum 1964; Bacigálová 2010
*T.viridis*								+				Grey-green lesions	Maire 1910; Mix 1949; Bacigálová 2010; This study
*Taphrina sp.1*		+										NA	UNITE
*Taphrina sp.2*										+		NA	GenBank

### ﻿Key to identification of alder colonising *Taphrina* in Northern hemisphere

**Table:** 

1	Infection symptoms develop on leaves; _D_-Glucitol and _L_-Lysine are assimilated	**2**
–	Symptoms appears as deformations of female catkins, _D_-Glucitol and _L_-Lysine are not assimilated	**5**
2	Symptoms in form of green round lesions on leaves, without any other plant deformities; melezitose assimilated, salicin weakly assimilated, _L_-arabinose and citrate not assimilated	**3**
–	Symptoms develop as leaf curl deformities; salicin assimilated, melezitose and _L_-arabinose not assimilated	**4**
3	Lesions on leaves grey-green; ascospores budding outside asci; asci small, in average 21.9–27.1 × 11–12.8 µm; nitrite assimilated, xylitol not assimilated, host *A.alnobetula*	***T.viridis***
–	Lesions on leaves regularly rounded, yellow; ascospores budding inside asci; nitrite not assimilated, xylitol assimilated; host mainly *A.glutinosa*, also known from A.×pubescens, *A.hirsuta* and *A.rugosa*	***T.sadebeckii***
4	Symptoms develop as leaf curls; ascospores budding outside asci; asci in average 25–33 × 8–15 µm; _L_-arabinose and _D_-mannitol assimilated, citrate, raffinose and melezitose not assimilated	***T.tosquinetii***
–	Symptoms are leaf curls but also witches-broom deformities; ascospores budding inside asci, asci bigger, in average 33–40 × 14–16 µm; _L_-arabinose and _D_-mannitol not assimilated, citrate, raffinose and melezitose assimilated	***T.epiphylla***
5	Asci supported with stalk cells; maltose and _L_-arabinose assimilated, _D_-glucitol not assimilated	***T.robinsoniana***
–	Asci without stalk cell; _D_-glucitol assimilated, maltose and _L_-arabinose not assimilated	***T.alni***

## ﻿Discussion

### ﻿Multilocus analysis confirmed the existence of *Taphrinaviridis* colonising *Alnusalnobetula*

While previous studies have conducted phylogenetic analyses incorporating alder-infesting *Taphrina* species (Rodrigues and Fonseca 2003; Inácio et al. 2004; Petrýdesová et al. 2013, 2016), the multilocus phylogeny presented here enables the first robust species delimitation within this group. Notably, it provides the initial molecular evidence establishing *T.viridis* as a distinct species colonising *A.alnobetula*. Search of the UNITE database also confirmed that there is only a single species colonising *A.alnobetula*, furthermore, that this species has never been found on any other hosts (Fig. 2). The key diagnostic features of *T.viridis* include host specificity to *A.alnobetula* and the presence of discoloured lesions ranging from yellow to brown. The descriptions of the microscopic structures of *T.viridis* in the literature largely agree with our observations (for details see Results), with the exception of stalk cells, which are notably smaller in Mix (1949) compared to those in Bacigálová (1994). These discrepancies could be attributable to the infraspecific variations caused by growth in different climatic and ecological conditions, however other explanations could also be variations in the presentation of statistical values and differences in the specific strains analysed in previous investigations (cf. Mix 1949; Bacigálová 1994, 2010; Bacigálová et al. 2003; Fonseca and Rodrigues 2011).

This species was widely overlooked and first distinguished from similar *T.sadebeckii* by Mix (1949), who studied only that type material of *T.alnastri* Lagerheim which he considered a conspecific species. As the original description of *Exoascusviridis* Sadeb. (Jaap 1901) is brief and does not allow us to identify the species with certainty, we designated a recent collection from *A.alnobetula* as the neotype to preserve its concept because of its distinct ecology. *Exoascus* is used to be a genus name for *Taphrina* anamorphs, and the combination *T.viridis* (Sadeb.) Maire was published more recently (Maire 1910) than *T.alnastri* (Vestergren 1903) and, as a result, it was meant to be a synonym for *T.viridis*, because they had the same host tree and the same symptomaticity. However, *T.viridis* has priority at the rank of species, since also *Exoascusviridis* was published at the same rank (cf. Rossman 2014). The original protologue in Jaap (1901) lacks any mention of collections or illustrations which could be considered as suitable original material. Therefore, we are not allowed to propose a lectotype and instead we must propose a neotype that fully aligns with the former species description of *T.viridis*. This case also emphasises the urgency of precise morphological and physiochemical descriptions with unified terminology, which serves as reliable sources and will allow unambiguous distinguishing of the respective taxa in the future.

The data on the occurrence of *T.viridis* have been limited to historical reports of the species from the Northern Alps in Germany (Jaap 1901; Vestergren 1903), the Alpes-Maritimes in France (Maire 1910) and more recent reports from the Western Carpathians in Slovakia (Bacigálová et al. 2003; Bacigálová 2010). Our search of the UNITE database (Abarenkov et al. 2024) was negative for this species. A search of GlobalFungi (Větrovský et al. 2020) yielded two records with a 100% match to the sequence of our strain BU 094. The first report concerned soil sample from the German Alps where *A.alnobetula* was present (Dahl et al. 2019). The second match originated from aerial and snow samples from the Austrian Alps at an elevation of 3106 m (Els et al. 2019). In summary, based on the currently available data, *T.viridis* is known to be present only in the higher mountain environments of the Alps and the Western Carpathians. The host tree *A.alnobetula*, in Europe, typically grows at higher elevations between 1660–2300 m and in addition to Alps and Carpathians, it occurs also in Apennines and Dinaric Mts. In addition, three taxa of this species are distributed in Siberia, North Europe, northwest North America and Japan (Mauri and Caudullo 2016). However, to date, there have been no reports of *Taphrina* infection or isolation of strains from *A.viridis* in the regions mentioned above.

An intriguing aspect arises concerning the ecology, specifically the host linkage, of *T.viridis*. While most alder-colonising *Taphrina* species exhibit a broad host range and have been documented on various alder species within the subgenus Alnus (refer to Table 2), *T.viridis* demonstrates a strict association with *A.alnobetula*. There are several potential reasons for this phenomenon, ranging from limited data and research on these parasitic fungi to the specific genomic properties that restrict its adaptation to particular host plants (cf. Wang et al. 2020). The strong host specificity observed in *T.viridis* may be influenced by the phylogenetic distance of *A.alnobetula* from its relatives, as this species belongs to the subgenus Alnobetula, unlike other European species which belong to the subgenera *Clethropsis* and *Alnus* (Chen and Li 2004; Ren et al. 2010). Additionally, we cannot rule out the possibility that the species has adapted to the hosts which occur in much colder mountain environments characterised by long-term low temperatures and shorter vegetation periods, as observed in other cold-adapted species such as *T.antarctica* and *T.gei-montani* which parasitize on the hosts plants in arctic alpine habitats (Selbmann et al. 2014; Petrýdesová et al. 2013, 2016).

### ﻿Unexpected species diversity of *Taphrina* parasitizing on alders inferred from environmental sequences

ITS analysis, which included all available sequences, for the first time indicated *Alnus* colonizing *Taphrina* species as a monophyletic group. Previous studies lacked this support, although they indicated a rather close relationship between alder colonising *Taphrina* species (Fonseca and Rodrigues 2011; Petrýdesová et al. 2013). However, it must be acknowledged that our analysis did not encompass the majority of other *Taphrina* species, which could potentially impact the outcomes of the analysis. Thus, the question of the monophyly of alder-parasitizing species warrants further substantiation through analyses that utilise comprehensive multilocus datasets, ideally encompassing the majority, if not all, of the recently accepted taxa.

The examination of sequences deposited in the UNITE and GlobalFungi databases, together with the compilation of all accessible data on alder-infecting species, revealed two notable findings. The first was the presence of two distinct groups of ITS sequences in the databases, that are indicative of undescribed and/or genetically not delimited species. The first taxon was identified in soil samples with presence of the European *A.glutinosa*. It forms, together with *T.tosquinetii* and *T.sadebeckii*, a trio of species which parasitize this single host species (Mix 1949; Bacigálová et al. 2003; Rodrigues and Fonseca 2003). The second comprised two sequences, one from *A.serrulata* in North America, and the other was purposedly isolated from *Betulaintermedia* in Sweden (CBS 417.54; GenBank accession number AF492079). This latter finding suggests a potential expansion of the host range from the *Alnus* species to the genus *Betula* (Betulaceae). However, our understanding of this isolate remains limited, and notably, Rodrigues and Fonseca (2003), who published this sequence, observed significant differences from other sequences originating from *Betula*, raising the possibility of mislabelling or misidentification of the host. Unfortunately, in all cases, we lacked the corresponding strains with which to perform comprehensive genetic, morphological, and biochemical analyses, as well as with which to observe and describe symptoms on infected trees, which is considered good practice in recent *Taphrina* taxonomy (cf. Rodrigues and Fonseca 2003; Inácio et al. 2004; Fonseca and Rodrigues 2011; Petrýdesová et al. 2013, 2016). Nevertheless, we view this finding as a significant milestone in *Taphrina* taxonomy and systematics, as database searches open new avenues for the identification of previously unrecognised entities. This direction allows scientists focused on this group to zoom in on specific species and the regions where such novel species are likely to occur.

## Supplementary Material

XML Treatment for Taphrina viridis

## ﻿Additional information

### Author ORCIDs

Michaela Caboňová https://orcid.org/0000-0002-2207-6183

Renáta Vadkertiová https://orcid.org/0000-0002-1401-5604

Slavomír Adamčík https://orcid.org/0000-0003-2156-5767

Marek Slovák https://orcid.org/0000-0001-9917-3099

Miroslav Caboň https://orcid.org/0000-0002-2255-3816

### Data availability

All of the data that support the findings of this study are available in the main text or Supplementary Information.

## Supplementary materials

Supplementary material 1 Comparison of morphological characteristics and physiological properties of *Alnus*-colonizing *Taphrina* species Data type pdf Explanation note Characteristics of species indicated with “1” are original to this study. Physiological properties of species labelled with “2” are adopted from Rodrigues and Fonseca (2011). Morphological characteristics indicated with “3” are adopted from Bacigálová (2010) and indicated with “4” are adopted from Mix (1949). Abbreviations used for description of physiological properties: + detected growth/utilisation; - not detected growth/utilisation; W weak growth/utilisation; V variable results; N not analysed; S slow growth/utilization. https://binary.pensoft.net/file/1130628This dataset is made available under the Open Database License (http://opendatacommons.org/licenses/odbl/1.0/). The Open Database License (ODbL) is a license agreement intended to allow users to freely share, modify, and use this Dataset while maintaining this same freedom for others, provided that the original source and author(s) are credited.Michaela Caboňová, Renáta Vadkertiová, Slavomír Adamčík, Kamila Bacigálová, Marek Slovák, Shanza Zaib1, Miroslav Caboň

## References

[B1] Abarenkov K Nilsson RH Larsson KH Taylor AFS May TW Frøslev TG Pawlowska J Lindahl B Põldmaa K Truong C Vu D Hosoya T Niskanen T Piirmann T Ivanov F Zirk A Peterson M Cheeke TE Ishigami Y Jansson AT Jeppesen TS Kristiansson E Mikryukov V Miller JT Oono R Ossandon FJ Paupério J Saar I Schigel D Suija A Tedersoo L Kõljalg U (2024) The UNITE database for molecular identification and taxonomic communication of fungi and other eukaryotes: Sequences, taxa and classifications reconsidered. Nucleic Acids Research 52(1): 791–797. 10.1093/nar/gkad1039 PMC10767974 37953409

[B2] Alonso A Pérez J Monroy S López-Rojo N Basaguren A Bosch J Boyero L (2021) Loss of Key Riparian Plant Species Impacts Stream Ecosystem Functioning. Ecosystems (New York, N.Y.) 24(6): 1436–1449. 10.1007/s10021-020-00592-7

[B3] Arhipova N Gaitnieks T Donis J Stenlid J Vasaitis R (2011) Decay, yield loss and associated fungi in stands of grey alder (Alnusincana) in Latvia. Forestry. Forestry 84(4): 337–348. 10.1093/forestry/cpr018

[B4] Bacigálová K (1994) Species of Taphrina on Alnus in Slovakia. Czech Mycology 47(3): 223–236. 10.33585/cmy.47308

[B5] Bacigálová K (2010) Flóra Slovenska X/2. VEDA vydavateľstvo Slovenskej akadémie vied, Bratislava, 184 pp.

[B6] Bacigálová K Lopandic K Rodrigues MG Fonseca A Herzberg M Pinsker W Prillinger H (2003) Phenotypic and genotypic identification and phylogenetic characterisation of Taphrina fungi on alder. Mycological Progress 2(3): 179–196. 10.1007/s11557-006-0056-1

[B7] Caboň M Li GJ Saba M Kolařík M Jančovičová S Khalid AN Moreau PA Wen HA Pfister DH Adamčík S (2019) Phylogenetic study documents different speciation mechanisms within the Russula globispora lineage in boreal and arctic environments of the Northern Hemisphere. IMA Fungus 10(5): 1–16. 10.1186/s43008-019-0003-9 32647614 PMC7325667

[B8] Chen Z Li J (2004) Phylogenetics and biogeography of Alnus (Betulaceae) inferred from sequences of nuclear ribosomal DNA ITS region. International Journal of Plant Sciences 165(2): 325–335. 10.1086/382795

[B9] Christita M Sipilä TP Auzane A Overmyer K (2022) Distinct Taphrina strains from the phyllosphere of birch exhibiting a range of witches’ broom disease symptoms. Environmental Microbiology 24(8): 3549–3564. 10.1111/1462-2920.16037 35579036 PMC9545635

[B10] Cissé OH Almeida JMGCF Fonseca A Kumar AA Salojärvi J Overmyer K Hauser PM Pagni M (2013) Genome Sequencing of the Plant Pathogen Taphrina deformans, the Causal Agent of Peach Leaf Curl. mBio 4(3): e00055–e13. 10.1128/mBio.00055-13 PMC3648899 23631913

[B11] Dahl MB Brejnrod AD Russel J Sørensen SJ Schnittler M (2019) Different degrees of niche differentiation for bacteria, fungi, and myxomycetes within an elevational transect in the german Alps. Microbial Ecology 78(3): 764–780. 10.1007/s00248-019-01347-1 30903202

[B12] Douda J Boublík K Slezák M Biurrun I Nociar J Havrdová A Doudová J Aćić S Brisse H Brunet J Chytrý M Claessens H Csiky J Didukh Y Dimopoulos P Dullinger S FitzPatrick Ú Guisan A Horchler PJ Hrivnák R Jandt U Kącki Z Kevey B Landucci F Lecomte H Lenoir J Paal J Paternoster D Pauli H Pielech R Rodwell JS Roelandt B Svenning JC Šibík J Šilc U Škvorc Ž Tsiripidis I Tzonev RT Wohlgemuth T Zimmermann NE (2016) Vegetation classification and biogeography of European floodplain forests and alder carrs. Applied Vegetation Science 19(1): 147–163. 10.1111/avsc.12201

[B13] Els N Larose C Baumann-Stanzer K Tignat-Perrier R Keuschnig C Vogel TM Sattler B (2019) Microbial composition in seasonal time series of free tropospheric air and precipitation reveals community separation. Aerobiologia 35(4): 671–701. 10.1007/s10453-019-09606-x

[B14] Fonseca A Rodrigues MG (2011) Taphrina Fries (1832). In: Kurtzman CP Fell JW Boekhout T (Eds) The Yeasts, a Taxonomic Study, 5th edn.Elsevier, Amsterdam, 823–858. 10.1016/B978-0-444-52149-1.00073-2

[B15] Gjaerum HB (1964) The genus Taphrina in Norway. Nytt Magasin for Botanikk 11: 5–26.

[B16] Gouy M Guindon S Gascuel O (2010) SeaView version 4: A multiplatform graphical user interface for sequence alignment and phylogenetic tree building. Molecular Biology and Evolution 27(2): 221–224. 10.1093/molbev/msp259 19854763

[B17] Inácio J Rodrigues MG Sobral P Fonseca A (2004) Characterisation and classification of phylloplane yeasts from Portugal related to the genus Taphrina and description of five novel Lalaria species. FEMS Yeast Research 4(4–5): 541–555. 10.1016/S1567-1356(03)00226-5 14734035

[B18] Jaap O (1901) Ein kleiner Beitrag zur Pilzflora von Tirol. Deutsche Botanische Monatsschrift 19(5): 74–77.

[B19] Katoh K Standley DM (2013) MAFFT multiple sequence alignment software, version 7: Improvements in performance and usability. Molecular Biology and Evolution 30(4): 772–780. 10.1093/molbev/mst010 23329690 PMC3603318

[B20] Katoh K Rozewicki J Yamada KD (2019) MAFFT online service: Multiple sequence alignment, interactive sequence choice and visualization. Briefings in Bioinformatics 20(4): 1160–1166. 10.1093/bib/bbx108 28968734 PMC6781576

[B21] Kearse M Moir R Wilson A Stones-Havas S Cheung M Sturrock S Buxton S Cooper A Markowitz S Duran C Thierer T Ashton B Meintjes P Drummond A (2012) Geneious Basic: An integrated and extendable desktop software platform for the organization and analysis of sequence data. Bioinformatics 28(12): 1647–1649. 10.1093/bioinformatics/bts199 22543367 PMC3371832

[B22] Kiran M Caboň M Senko D Khalid AN Adamčík S (2021) Description of the fifth new species of Russula subsect. Maculatinae from Pakistan indicates local diversity hotspot of ectomycorrhizal fungi in Southwestern Himalayas. Life (Basel, Switzerland) 11(7): 662–676. 10.3390/life11070662 34357034 PMC8303804

[B23] Kramer CL (1987) The Taphrinales. In: de Hoog GS Smith MT Weijman ACJ (Eds) The Expanding Realm of Yeast-like fungi.Elsevier, Amsterdam, 151–166.

[B24] Kurtzman CP Fell JW Boekhout T Robert V (2011) Methods for isolation, phenotypic characterization and maintenance of yeasts. In: Kurtzman CP Fell JW Boekhout T (Eds) The Yeasts – A Taxonomic Study, 5th edn.Elsevier, London, 87–110. 10.1016/B978-0-444-52149-1.00007-0

[B25] Lanfear R Calcott B Ho SYW Guindon S (2012) PartitionFinder: Combined selection of partitioning schemes and substitution models for phylogenetic analyses. Molecular Biology and Evolution 29(6): 1695–1701. 10.1093/molbev/mss020 22319168

[B26] Maddison WP Maddison DR (2019) Mesquite: A modular system for evolutionary analysis. Version 3.61. Available online: http://mesquiteproject.org [accessed 18.4.2021]

[B27] Maire MR (1910) Contribution á l’etude de la Flore mycologique des Alpes-Maritimes. – Champignons récoltés á la Session de Saint- Martin- Vésubie. Bulletin de la Société Botanique de France 57: 166–176. 10.1080/00378941.1910.10839687

[B28] Mauri A Caudullo G (2016) Alnusviridis in Europe: distribution, habitat, usage and threats. In: San-Miguel-Ayanz J, de Rigo D, Caudullo G, Houston DT, Mauri A (Eds) European Atlas of Forest Tree Species. Publ. Off. EU, Luxembourg, 68 pp.

[B29] Miller M Pfeiffer W Schwartz T (2010) Creating the CIPRES Science Gateway for inference of large phylogenetic trees. Proceedings of the gateway computing environments workshop (GCE), New Orleans (USA), November, IEEE Publisher, 1–8. 10.1109/GCE.2010.5676129

[B30] Mix AJ (1949) A monograph of the genus Taphrina. The University of Kansas Science Bulletin 33(1): 3–167. 10.5962/bhl.part.16125

[B31] Mix AJ (1954) Additions and emendations to a monograph of the genus Taphrina. Transactions of the Kansas Academy Science 57: 55–65. 10.2307/3625642

[B32] Petrýdesová J Bacigálová K Sulo P (2013) The reassignment of three ‘lost’ Taphrina species (Taphrina bullata, Taphrina insititiae and Taphrina rhizophora) supported by the divergence of nuclear and mitochondrial DNA. International Journal of Systematic and Evolutionary Microbiology 63(8): 3091–3098. 10.1099/ijs.0.052712-0 23710051

[B33] Petrýdesová J Kučera J Bacigálová K Vadkertiová R Lopandic K Vďačný P Slovák M (2016) Disentangling identity of species of the genus Taphrina parasitizing herbaceous Rosaceae, with proposal of Taphrinagei-montani sp. nov. International Journal of Systematic and Evolutionary Microbiology 66(7): 2540–2549. 10.1099/ijsem.0.001095 27098204

[B34] Rambaut A Suchard M Xie D Drummond A (2014) Tracer. Version 1.6. http://beast.bio.ed.ac.uk/software/tracer/ [accessed 18.4.2021]

[B35] Rehner SA Buckley E (2005) A Beauveria phylogeny inferred from nuclear ITS and EF1-α sequences: Evidence for cryptic diversification and links to Cordyceps teleomorphs. Mycologia 97(1): 84–98. 10.3852/mycologia.97.1.84 16389960

[B36] Ren BQ Xiang XG Chen ZD (2010) Species identification of Alnus (Betulaceae) using nrDNA and cpDNA genetic markers. Molecular Ecology Resources 10(4): 594–605. 10.1111/j.1755-0998.2009.02815.x 21565064

[B37] Rodrigues MG Fonseca A (2003) Molecular systematics of the dimorphic ascomycete genus Taphrina. International Journal of Systematic and Evolutionary Microbiology 53(2): 607–616. 10.1099/ijs.0.02437-0 12710634

[B38] Rohrs-Richey JK Mulder CPH Winton LM Stanosz G (2011) Physiological performance of an Alaskan shrub (Alnus fruticosa) in response to disease (Valsa melanodiscus) and water stress. The New Phytologist 189(1): 295–307. 10.1111/j.1469-8137.2010.03472.x 20868393

[B39] Ronquist F Teslenko M Van Der Mark P Ayres DL Darling A Höhna S Larget B Liu L Suchard MA Huelsenbeck JP (2012) MrBayes 3.2: Efficient Bayesian phylogenetic inference and model choice across a large model space. Systematic Biology 61(3): 539–542. 10.1093/sysbio/sys029 22357727 PMC3329765

[B40] Rossman AY (2014) Lessons learned from moving to one scientific name for fungi. IMA Fungus 5(1): 81–89. 10.5598/imafungus.2014.05.01.10 25083410 PMC4107901

[B41] Salata B (1974) Grzyby (Mycota), vol. 6, Workowce (Ascomycetes), szpetkowe (Taphrinales). In: Kochman J et al. (Eds) Flora Polska, Polska Akademia Nauk, Warszawa-Krakow, 1–87.

[B42] Selbmann L Turchetti B Yurkov A Cecchini C Zucconi L Isola D Buzzini P Onofri S (2014) Description of Taphrinaantarctica f.a. sp. nov., a new anamorphic ascomycetous yeast species associated with Antarctic endolithic microbial communities and transfer of four Lalaria species in the genus Taphrina. Extremophiles 18(4): 707–721. 10.1007/s00792-014-0651-z 24893860

[B43] Stamatakis A (2014) RAxML version 8: A tool for phylogenetic analysis and post-analysis of large phylogenies. Bioinformatics 30(9): 1312–1313. 10.1093/bioinformatics/btu033 24451623 PMC3998144

[B44] Stöver BC Müller KF (2010) TreeGraph 2: Combining and visualizing evidence from different phylogenetic analyses. BMC Bioinformatics 11(7): 1–9. 10.1186/1471-2105-11-7 20051126 PMC2806359

[B45] Sulo P Laurenčík M Poláková S Minárik G Sláviková E (2009) Geotrichum bryndzae sp. nov., a novel asexual arthroconidial yeast species related to the genus Galactomyces. International Journal of Systematic and Evolutionary Microbiology 59(9): 2370–2374. 10.1099/ijs.0.008938-0 19605724

[B46] Vestergren JTC (1903) Micromycetes Rariores Selecti. Exsiccat no. 720 Taphrinaalnastri Lagerh.

[B47] Větrovský T Morais D Kohout P Lepinay C Algora C Awokunle Hollá S Bahnmann BD Bílohnědá K Brabcová V D’Alò F Human ZR Jomura M Kolařík M Kvasničková J Lladó S López-Mondéjar R Martinović T Mašínová T Meszárošová L Michalčíková L Michalová T Mundra S Navrátilová D Odriozola I Baldrian P (2020) GlobalFungi, a global database of fungal occurrences from high-throughput-sequencing metabarcoding studies. Scientific Data 7(1): 228. 10.1038/s41597-020-0567-7 32661237 PMC7359306

[B48] Vilgalys R Hester M (1990) Rapid genetic identification and mapping of enzymatically amplified ribosomal DNA from several Cryptococcus species. Journal of Bacteriology 172(8): 4239–4246. 10.1128/jb.172.8.4238-4246.1990 PMC213247 2376561

[B49] Wang Q Sun M Zhang Y Song Z Zhang S Zhang Q Xu JR Liu H (2020) Extensive chromosomal rearrangements and rapid evolution of novel effector superfamilies contribute to host adaptation and speciation in the basal ascomycetous fungi. Molecular Plant Pathology 21(3): 330–348. 10.1111/mpp.12899 31916390 PMC7036362

[B50] White TJ Bruns T Lee S Taylor J (1990) Amplification and direct sequencing of fungal ribosomal RNA genes for phylogenetics. In: Innis MA, Gelfand DH, Sninsky JJ, White TJ (Eds) PCR protocols a guide to methods and applications, Academic Press, San Diego, 315–322. 10.1016/B978-0-12-372180-8.50042-1

