# Supplementary material for: ﻿Taxonomic reintroduction of Taphrinaviridis (Taphrinales, Ascomycota) associated with Alnusalnobetula as one of five well defined European species colonizing alders
Source: MycoKeys. 2024 Sep 10;108:249–67. doi: 10.3897/mycokeys.108.127292 (PMC11408875; doi:10.3897/mycokeys.108.127292)
Supplement: Supplementary material 1 — Comparison of morphological characteristics and physiological properties of Alnus-colonizing Taphrina species [file mycokeys-108-249-s001.pdf]

Taxonomic reintroduction of *Taphrina viridis* (Taphrinales, Ascomycota) associated with *Alnus alnobetula* as one of five well defined European species colonizing alders

Caboňová Michaela[1], Vadkertiová Renáta[2], Adamčík Slavomír[1,3], Bacigálová Kamila[1], Slovák Marek[1,4], Zaib Shanza[1], Caboň Miroslav[1,5,*]

Supplementary Table 1: Comparison of morphological characteristics and physiological properties of Alnus-colonizing *Taphrina* species. Characteristics of species indicated with "[1]" are original to this study. Physiological properties of species labelled with "[2]" are adopted from Rodrigues and Fonseca (2011). Morphological characteristics indicated with "[3]" are adopted from Bacigálová (2010) and indicated with "[4]" are adopted from Mix (1949). Abbreviations used for description of physiological properties: + detected growth/utilisation; - not detected growth/utilisation; W weak growth/utilisation; V variable results; N not analysed; S slow growth/utilization

		**Viridis[1]**	**Sadebeckii[2,3]**	**Sadebeckii[1]**	**Epiphylla[2,3]**	**Alni[2,3]**	**Tosquinetii[2,3]**	**Robinsoniana[2,4]**
**Ascus**	Length	21.9–27.1	41–53		33–40	40–48	25–33	13–43
	Width	11–12.8	15–16		14–16	13–16	8–15	6–17
	Spores	8	8		8	8	8	8
**Ascospore**	Length	5.3–7.7	4–6		4–5	2.5–7.5	3–4	2.5–6
	Width	3.9–4.9	3.5–5		4–7	4–5	3–5	2.5–5
	Buding	Outside asci	Inside asci		Inside asci	Inside asci	Outside, occasionaly inside asci	Inside asci
**Stalk cells**	Length	12.4–17.3	8–16		8–10	NP	8–10	6–20
	Width	11.1–16.9	16–33		25–33	NP	10–16	5–17
**Physiological properties**	Glucose	+	+	+	+	V	+	+
	Inulin	-	+	-	W	-	-	-
	Sucrose	+	+	+	+	+	+	+
	Raffinose	-	V	w	+	-	-	-
	Melibiose	-	-	-	-	-	-	-
	Galactose	-	-	-	W	-	-	-
	Lactose	-	-	-	-	-	-	-
	Trehalose	-	-	-	-	-	S	-
	Maltose	-	-	-	-	-	S	+
	Melezitose	+	+	+	+	+	-	V

		Viridis[1]	Sadebeckii[2,3]	Sadebeckii[1]	Epiphylla[2,3]	Alni[2,3]	Tosquinetii[2,3]	Robinsoniana[2,4]
	Soluble starch	+	+	+	+	+	W	V
	Cellobiose	+	+	+	+	+	+	+
	Salicin	w	+	w	+	V	+	W
	L-Sorbose	-	-	-	-	-	-	-
	L-Rhamnose	-	-	-	-	-	-	-
	D-Xylose	+	+	+	+	+	+	+
	L-Arabinose	-	-	-	-	-	+	+
	D-Arabinose	-	-	-	-	V	-	-
	D-Ribose	-	-	-	W	-	-	-
	Methanol	-	-	-	-	-	-	-
	Ethanol	-	V	-	-	-	-/s	V
	Glycerol	+	+	+	+	+	+	+
	Erythritol	-	-	-	-	-	-	-
	Ribitol	-	-	-	-	-	-	-/w
	D-Mannitol	+	+	+	-	+	+	+
	D-Glucitol	+	V	+	+	+	+	-
	myo-Inositol	-	-	-	-	-	-	-
	DL-Lactate	-	-	-	-	-	-	-/w
	Succinate	w	+	+	+	+	+	+
	Citrate	-	+	-	+	+	-	+
	D-Glucosamine	-	-	-	-	-	-	-
	N-Acetyl-D-glucosamine	-	N	-	N	N	N	N
	Nitrate	+	+	+	+	+	+	+
	Vitamine-free	w	-	w	W	W	W	W
	Xylitol	-	+	+	+	V	S	V
	Nitrite	+	-	-	-	-	-	-
	Ethylamine	-	-	-	-	-	-	V
	L-Lysine	+	V	+	+	-	+	+/w
	Starch formation	+	+	+	+	+	+	+
	Urea hydrolysis	+	+	+	+	+	W	+
	DBB	-	-	-	-	-	-	-
	Fermentation	absent	absent	absent	absent	absent	absent	absent
